# Enormous gastrointestinal stromal tumor arising from the stomach causing weight loss and anemia in an elderly female: Case report

**DOI:** 10.1016/j.ijscr.2019.09.045

**Published:** 2019-10-10

**Authors:** Ayad Ahmad Mohammed, Sardar Hassan Arif

**Affiliations:** Department of Surgery, College of Medicine, University of Duhok, Kurdistan Region, Iraq

**Keywords:** Gastrointestinal stromal tumors, GIST, Interstitial cell of Cajal, CD117, c-kit protein, Imatinib

## Abstract

**Introduction:**

Gastrointestinal stromal tumors are the most common mesenchymal tumors of gastrointestinal tract originating from the interstitial cell of Cajal. The presentation is variable depending on the size and site. CT scan is the most widely used imaging. The primary treatment is surgical resection. Imatinib may be used in unresectable tumors or preoperatively.

**Case presentation:**

A 65-year-old lady had weight loss and repeated vomiting for one year. Examination showed a non-tender mass in the left hypochonrdium arising below the costal margin extending to 15 cm toward the left iliac fossa. It was firm with smooth surface. CT-scan examination showed a 45 cm × 21 cm mass arising between the spleen and the left kidney and displacing the kidney toward the midline. Gastroscopy and biopsy showed gastrointestinal stromal tumor. During surgery an enormous gastric tumor attached to the spleen. Total gastrectomy en-block with splenectomy and reconstruction done through roux-en-y esophago-jejunostomy and jejunojejunostomy.

**Conclusion:**

GISTs most commonly affect the stomach, most tumors are treated by primary surgical excision with normal safety margins, advanced disease may need imatinib before surgery which helps in decreasing the size of the tumor and makes surgery easier.

## Highlights

• Gastrointestinal stromal tumors are the most common mesenchymal tumors of gastrointestinal tract.
• Surgical excision with normal safety margins is the main form of treatment.
• Preoperative imatinib therapy is used in unresectable or recurrent tumors.
• Huge tumors may not respond to medical therapy.

## 1 Introduction

Gastrointestinal stromal tumors (GISTs) are the most common tumors of mesenchymal origin that affect the gastrointestinal tract(GIT) [1].

These tumors are rare and constitutes about 0.1–0.3% of all malignancies affecting the GIT. The vast majority of these tumors affect the stomach (60%) and the rest affect other parts of the GIT like the colon, rectum, esophagus and the appendix in order of decreasing frequency. The primary tumor is usually single but sometimes may be multiple, recurrent tumor usually involves multiple sites [1,2].

GISTs originate from the interstitial cell of Cajal or the precursors of this cell and occur due to mutation in the TKI and the platelet derived growth factor receptors. They consists of consists of spindle cells or epithelioid cells or both types of cells in rare occasions. The tumors express CD117 (c-kit protein) [3,4].

The gross pathological appearance is submucosal lesion that appears to arise from the muscular layer of the bowel that may show intraluminal of extramural pattern of growth [1].

The clinical presentation is variable depending on the size of the tumor and the anatomical site. Some tumors are discovered accidentally during surgery for other pathologies, some patients may present with chronic epigastric pain, early satiety, acute or chronic GIT bleeding. Emergency presentations are uncommon but may include perforation, intestinal obstruction, and peritonitis. The emergency surgeon should follow the surgical guidelines for managing such tumors such as complete resection of the tumor and avoiding capsule rupture when intact [1,2].

CT scan is the most widely used imaging modality which can detect the majority of the lesions, it can detect the accurate size, and the anatomical relations with other adjacent organs [5].

The risk of tumor recurrence is categorized according to the Fletcher criteria, this categorization is depending on the size of the primary tumor and the mitotic rate. In this classification the author classifies the tumors into 4 main groups: very low risk of recurrence group when the size of the tumor is less than 2 cm and the mitotic rate is less than 5 mitotic counts per 50 high power fields(HPF), low risk group when the size of the tumor is between 2–5 cm and the mitotic count is less than 5 per HPF, intermediate risk group when the tumor size is less than 5 cm and between 6–10 mitotic counts per HPF or tumor size between 5–10 cm and less than 5 mitotic counts per HPF, and the high risk group patients when the tumor size is more than 5 cm with more than 5 mitotic counts per HPF or tumor size more than 10 cm with any mitotic count or the mitotic count is more than 10 per HPF regardless of the size [6].

The work of this case report has been reported in line with the SCARE criteria 2018 [7].

## 2 Patient information

A 65-year-old lady presented with weight loss and repeated attacks of vomiting for the last year. The vomiting was occurring after the meals and was non projectile and non-bilious with no blood. The patient reported history of loss of 15 kg within this period. The patient had negative surgical history, she had hypertension controlled with diet and medications. The family history was negative for malignancies.

### 2.1 Clinical findings

During examination she was pale with no jaundice, the vital signs were normal and the abdominal examination showed a non-tender mass in the left hypochonrdium. The mass was arising from below the left costal margin and extended to 15 cm toward the left iliac fossa. It was firm in consistency with smooth surface. The mass showed no mobility.

### 2.2 Diagnostic assessment

The blood tests showed low hemoglobin level (9 g/dl), with normal white blood cell count, and platelets. The renal function was normal.

**Fig. 1:**
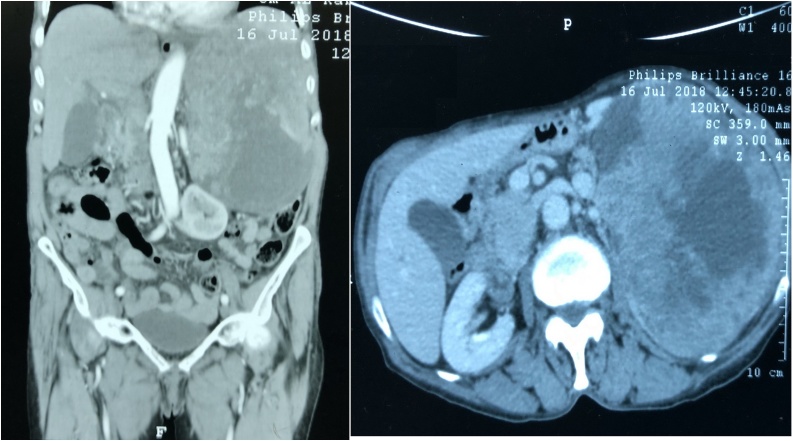
CT scan of the abdomen showing a huge mass that occupy the left upper abdominal cavity and displacing the left kidney, the mass has mixed cystic and solid components.

Ultrasound and CT-scan examinations of the abdomen showed a huge mass about 45 cm × 21 cm arising from the area between the spleen and the left kidney and displacing the kidney toward the midline. The mass has areas of cystic and solid components. The possible origin was from the gastric wall and they advised biopsy for confirmation Fig. 1.

Gastroscopy and biopsy showed a huge mass that compressing the gastric cavity with no areas of ulceration, biopsy taken.

The results of the biopsy showed gastrointestinal stromal tumor.

### 2.3 Therapeutic intervention

Neoadjuvant imatinib, a TKI tyrosine kinase inhibitor, started in the hope of decreasing the tumor size with no any response.

**Fig. 2:**
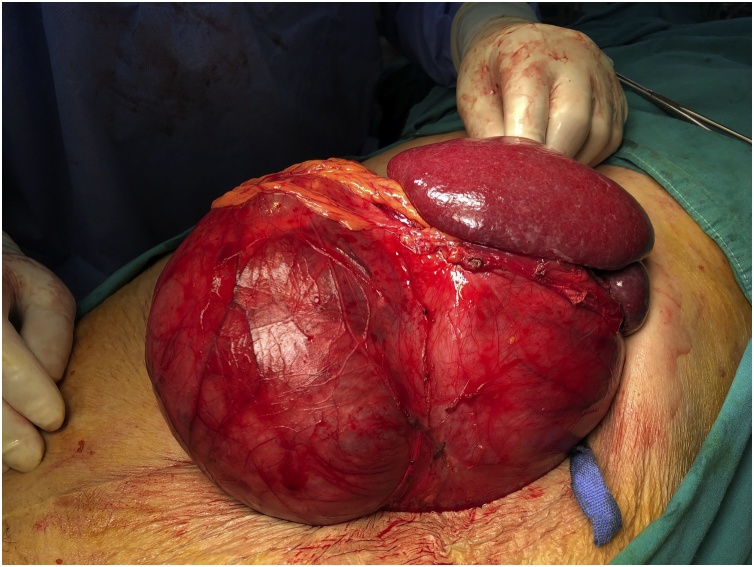
Intraoperative picture showing the huge gastric GIST tumor with the spleen.

Surgery done through midline incision, an enormous mass found arising from the gastric wall and attached to the spleen. The tumor caused displacement of the left kidney to the region of the duodeno-jejunal flexure (Fig. 2).

Total gastrectomy en-block with splenectomy and reconstruction done through roux-en-y esophago-jejunostomy and jejunojejunostomy. The histopathological examination confirms the diagnosis GIST tumor with 5 mitotic counts per HPF.

### 2.4 Follow-up and outcomes

The patient discharged home after one week with no postoperative complications. The patient is categorized in the very high risk group for recurrence we are planning to treat the patient with the adjuvant TKI 400 mg daily for the next 3 years with follow up Ct scans for the detection of early recurrence or metastatic disease.

## 3 Discussion

Because of the vague symptoms that are caused by this tumor, the disease is discovered when the tumor is large in size that cause pressure effects or increasing pain intensity, small tumors are usually discovered incidentally which accounts for about one third of the cases. Spontaneous rupture is uncommon but when rupture occurs it usually occurs to the lumen of the bowel causing bleeding to the lumen [1].

The tumor size and the mitotic rate are the most common criteria that determine the prognosis of such types of tumor. The principal ways of tumor spread are usually locally and to the liver, involvement of the regional lymphatics is rare [2,8,9].

The presence of metastatic lesions in other anatomical places represents a late stage of the disease and this event cannot be controlled by imatinib alone [9].

Surgery is still the gold standard modality of treatment involving radical resection of the tumor with normal margins of surrounding tissue, however in some patients the tumor cannot be excised completely due to its difficult anatomical site like the gastroesophageal junction, the duodenum, and the lower rectum, especially if the tumor is very large in size. Such surgery is usually associated with higher morbidity and mortality. During surgery it is important to avoid rupture of the capsule of the tumor and intra-abdominal spillage of the tumor cells to decrease the recurrence rate [3,8,10].

Surgery can be done by the conventional open technique or the laparoscopic technique, Laparoscopic surgery can be done when the tumor small in size but will be difficult for large size tumors [8].

Specific tyrosine kinase receptor inhibitors such as imatinib is a type of targeted therapy that inhibit the growth activity of the tumor. Preoperative imatinib therapy have been shown to be effective in unresectable or recurrent tumors, it causes reduction in the tumor size which makes subsequent surgery easier and the patient have better clinical outcome [3,4].

The survival rate after successful surgical resection varies between 48–80%, when the resection is not complete less than 10% of patients survives beyond 1 year [1].

Most authors advocate careful and regular follow up and for indefinite period of time [1].

## Sources of funding

None.

## Ethical approval

Ethical approval has been exempted by my institution for reporting this case.

## Consent

Written informed consent was obtained from the patient for publication of this case report and accompanying images.

## Author’s contribution

Dr Ayad Ahmad Mohammed and Dr Sardar Hassan Arif are the surgeons who performed the operation.

The concept of reporting the case, data recording, and drafting the work done by Dr Ayad Ahmad Mohammed and Dr Sardar Hassan Arif.

Dr Sardar Hassan Arif took the consent from the patient for publishing the case.

Final approval of the work to be published was done by Dr Ayad Ahmad Mohammed.

## Registration of research studies

This work is case report and there is no need of registration.

## Guarantor

Dr Ayad Ahmad Mohammed is guarantor for the work.

## Provenance and peer review

Not commissioned, externally peer-reviewed.

## Declaration of Competing Interest

The author has no conflicts of interest to declare.
